# Vertical Trapping of the Coffee Berry Borer, *Hypothenemus hampei* (Coleoptera: Scolytinae), in Coffee

**DOI:** 10.3390/insects12070607

**Published:** 2021-07-02

**Authors:** Claudia Patricia Ruiz-Diaz, José Carlos Verle Rodrigues

**Affiliations:** Center for Excellence in Quarantine & Invasive Species, Agricultural Experimental Station—Río Piedras, Department of Agroenvironmental Sciences, University of Puerto Rico—Mayagüez, 1193 Calle Guayacán, San Juan, PR 00681, USA; claudia.ruiz2@upr.edu

**Keywords:** *Coffee arabica*, *Hypothenemus hampei*, baited traps, IPM

## Abstract

**Simple Summary:**

Globally, the Coffee Berry Borer, *Hypothenemus hampei* (Ferrari) (Coleoptera: Curculionidae: Scolytinae), severely affects the quality and production of coffee. To understand how to control this pest, we studied their capture patterns at different trapping heights over time. Baited column traps captured more coffee berry borers at 0.5m height than at higher heights irrespective of temperature changes, rainfall, and relative humidity. Understanding the height position for the most efficient insect capture is useful for developing future cost-effective management strategies to control this coffee pest.

**Abstract:**

The coffee industry loses millions of dollars annually worldwide due to the Coffee Berry Borer (CBB); these losses imply a decrease in quality and production. Traps are used to monitor their flight and for pest control. The main objective of this study was to determine the capture pattern and trap capture percentages of the CBB population over time using column traps (CTs) in two independent field experiments. CTs were composed of four traps installed at four different heights 0.5, 1.5, 2.5, and 3.5 m above ground. Our results demonstrated a significant difference in CBB capture by traps placed at different heights above the ground. The CT capture maintained a pattern throughout this study’s lag: the lower the height, the greater the percentage of CBBs captured. The study was conducted in two independent experiments (A and B). In Experiment A and B, the traps placed at 0.5 m caught 67% and 85% of the CBBs captured, respectively. Furthermore, the trap set at 1.5 m above the ground in the multi-level CT showed a higher capture percentage than the single placed trap (ST, also at 1.5 m about ground). The pattern of the capture and proportion of the CBB in the CTs was maintained throughout the study despite the season, changes in temperature, and relative air humidity. We suggest that CTs could be explored as a useful tool for capturing the CBB, considering its monitoring and management.

## 1. Introduction

The Coffee Berry Borer (CBB), *Hypothenemus hampei* (Ferrari, 1867) (Coleoptera: Scolytidae), is a major invasive species in coffee [1,2]. Trapping is one of the most traditional and well-known integrated pest management (IPM) strategies on coffee plantations worldwide for monitoring and pest control [3,4,5,6,7,8]. Alcohol-baited traps are primarily used to monitor flight activity and control the CBB [1,3,9,10]. The CBB uses the coffee berries for shelter, feeding, and reproduction, causing a decrease in coffee bean quality and production [6,11]. Around the world, the coffee industry loses more than US $500 million annually as a consequence of CBB damage [12]. In Puerto Rico (PR), in some locations, CBB infestation reached up to 95% during the 2014 season, causing total crop loss [13].

A relationship has been reported between the number of CBBs captured by traps baited with a methanol: ethanol solution and the infestation of the coffee berries [5,8,14,15,16]. Different trap designs and proportions of ethanol and methanol have been tested worldwide in search of a combination that captures the most significant number of CBBs [3,5,10,14,15,16,17]. In most trapping designs, the traps are placed at a height of between 1.5 m and 2.0 m with a single unit and a bait container of 17 to 40 mL with a 3:1 solution methanol: ethanol [5,10,14,18,19,20]. Further to this, the rate of emission of the solution is essential to attract and capture CBBs. In 1997, Mathieu et al. [3] suggested that the trap’s emission rate is inversely proportional to the number of CBB captures for a methanol: ethanol mixture in funnel traps. Mathieu and collaborators recommended that a single trap might be a better control option for the CBB than other designs.

**Figure 1:**
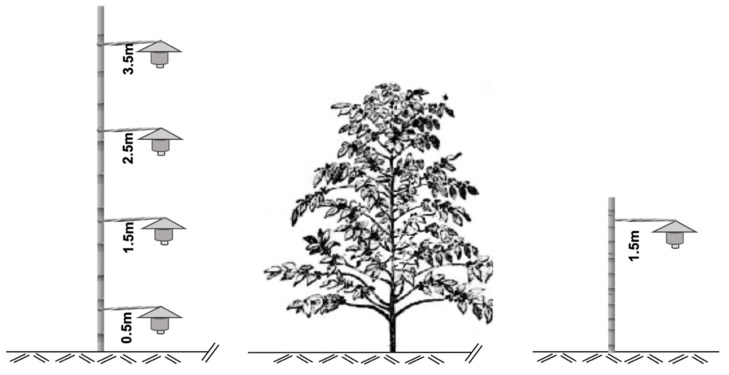
Representative illustration of column traps (CT). Each CT comprised of four traps placed at 0.5 m, 1.5 m, 2.5 m, and 3.5 m above ground and a single trap (ST) at 1.5 m above ground. The field experiments were conducted in Adjuntas, Puerto Rico.

Wind, rain, temperature, and availability of the berries directly influence the flight range of the CBBs [15,16]. For a trapping system, several questions remain about interactions among the characteristics and behavior of the CBB, climate factors, and the density and phenology of the coffee plants. How much the insect will disperse and move horizontally or vertically is important to improving CBB control and monitoring. What would be the ideal height to capture the largest number of CBBs [10,15,16,20]? The main objectives in this work were: (1) to establish the relationship between field infestation and capture by the traps, and (2) to determine the capture of the CBB by standard Brocap^®^ traps installed in columns at four different heights (0.5 m, 1.5 m, 2.5 m, and 3.5 m), over distinct stages of the coffee phenology and weather periods (Figure 1). 

## 2. Materials and Methods

### 2.1. Study Sites

The field experiments were conducted at the Agricultural Experiment Station of Adjuntas, PR (18 10′28.89″ N 66 47′ 52.27″ W) at 585 m above sea level (masl) in *Coffee arabica* “Limani” and “Catuaí”. Coffee plants averaged 2.5 m in height in the study plots and were separated at 1.80 m × 1.50 m (inter-row x plants within the row). The three plots used in this study were cultivated in monoculture and in full sun conditions. The main blossoms came between February and March and the coffee harvest was carried out by hand-picking between the months of October and December. The harvest was done only for the mature berries and multiple flowering during the season provided a constant presence of the fruits. Experiment A (Exp A, “Limani”) was conducted between April and September 2019 in the first plot, which has an area of 2280 m^2^; Experiment B (Exp B, “Catuaí”), conducted between October 2019 and March 2020, was established in a second plot that has an area of 2800 m^2^. In the referential coffee plot (Referential 1 and 2 - Ref 1 and 2) with an area of 1200 m^2^, single traps (Brocap^®^, CBB trap CIRAD) at 1.5 m height were installed (Figures S1 and S2). The column traps (CTs) and single traps (STs) were placed at 1 m on the coffee trees. Meteorological data were recorded by a Remote Monitoring Weather Station Data Logger HOBO RX3000 (Onset, Bourne, MA, USA).

### 2.2. Column Trap Designed

The CT design held four traps (Brocap^®^) placed at four heights above ground. Five CTs (repetitions) were positioned 25 m from each other in a zig-zag pattern in each plot, and at 30 cm from the tree canopy. Each trap contained a 30 mL container with a chemical lure (3:1, methanol: ethanol). The lure was replaced every six weeks or sooner if it was observed to have evaporated. The traps were fixed one above the other at 3.5 m, 2.5 m, 1.5 m, and 0.5 m above the ground; and five STs at 1.5 m above the ground were placed at the reference plot (Figure 1 and Figure S1). The same trap design and disposition were used in all experiments.

### 2.3. Data Collection

All of the data were collected from two separate experiments and two referential coffee plots; the data were collected biweekly (see Table 1). Exp A and Ref 1 were carried out from March to September 2019, and Exp B and Ref 2, from October 2019 to March 2020. In each sampling, all the CBBs captured were duly separated and identified by the number of the CT and its corresponding height. Then, the samples were taken to the laboratory to identify and quantify. For the infestation, we first randomly selected three branches from trees around each CT and ST, recorded the total number of berries per coffee branch (each branch chosen had a minimum of 33 berries), and counted how many perforated berries were on the branch for a total of fifteen branches per plot for each sampling date. The same was done for the reference coffee plot (Ref 1 and Ref 2).

### 2.4. Data Analysis

The CBB capture rate pattern was monitored by the height of each of the traps in the CTs. We also monitored the percentage of infestation of the coffee berries in each plot. The percentage of infestation was calculated by the ratio of perforated berries to whole berries. To fit a normal distribution, the data were transformed according to Warton [21]. The CBB capture rate per trap was normalized per day and transformed according to [21]. Furthermore, we used the proportions of CBB capture at each height over the total number of CBBs captured by the CTs to analyze the capture pattern at each height over time. Such analysis was done with a generalized linear model with suitable distribution function links. The CBB catch per height over time was analyzed using a generalized linear model with log link gamma function. The response variable was CBB catch per height per sampling, with the explanatory variables of time, trap height, and its iteration. To evaluate data normality, we used the Shapiro-Wilk test and Spearman’s rank correlation test for infestation and CBB capture rate by CT. For each experiment, Exp A and Exp B were analyzed separately, given the temporal and spatial differences. The statistical analysis was performed using RStudio Team V1.2.1335, and for figures, we used the packages ggplot2 and MASS for glm analysis [22,23,24,25].

## 3. Results

**Figure 2:**
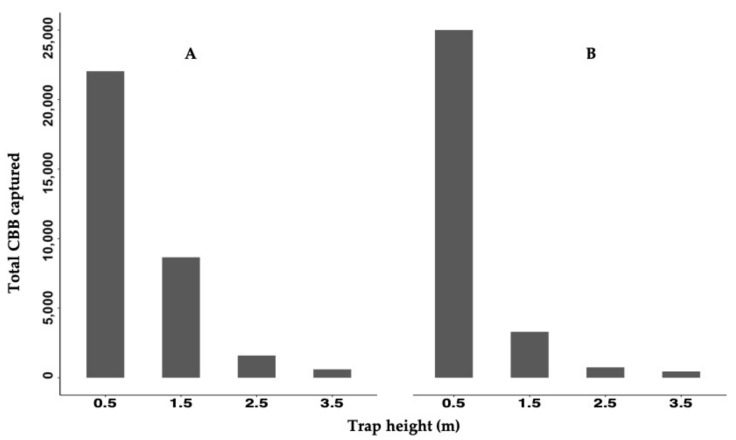
The total CBB captured at each height in each experiment. Experiment **A** was established from 2 March to 4 September 2019 and Experiment **B** from 2 October to 4 March 2020 in Adjuntas, Puerto Rico.

A total of 72,172 CBBs were captured using CTs during the periods of the two studies: Table 1 shows there were 32,899 CBBs in Exp A and 39,273 in Exp B. In addition, 7635 CBBs in Ref 1 and 2665 in the Ref 2 were captured by single traps at the reference plot. The traps placed at 3.5 m and 2.5 m height were the ones that collected the least CBBs, and the most significant collection of CBBs was at 0.5 m, followed by 1.5 m in height. A similar pattern could be observed at the two study sites and in all CTs (Figure 2). The trap placed at 0.5 m height collected 67.0% and 84.7% of the total CBBs for Exp A and Exp B, respectively. The trap at 1.5 m collected 26.3% and 11.2%, in Exp A and Exp B, respectively. The traps at 2.5 m and 3.5 m combined represented 6.7% in Exp A and 4.1% in Exp B (Figure 2). The environmental variables collected during the two experiments are summarized in Table 1. The averages of temperature, relative humidity, total rainfall, rain events, and dry days were assessed. The second experiment was conducted during a drier period though it had more events of rain.

**Figure 3:**
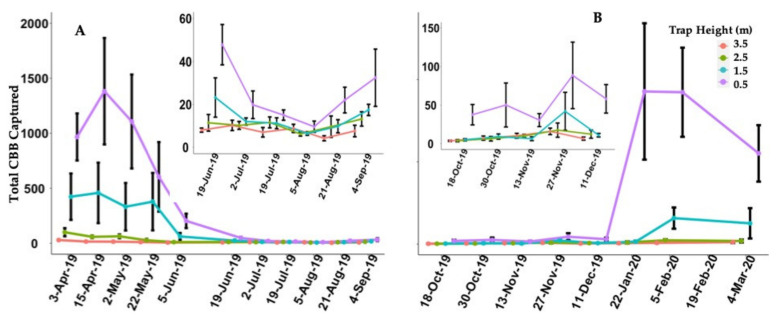
(**A**) Average and standard error per collection date for each height of the Column Traps over the time of Experiment A. (**B**) Average and standard error per collection date for each height over the time of Experiment B, Adjuntas, Puerto Rico. At the top, there is a close-up of CBB catches in the period when the catch decreases. However, the Column Traps follow the same pattern of catches by height: the lower the height, the greater the CBB catch.

Results from general linear model for Exp A showed that the percentage of each trap’s capture, according to height for the total CBB captured by column, was statistically different by height over time with *χ^2^* = 177.1, *df* = 43, *P-value* = 0.001. These results show that the relative difference in the CBB captured per height over time was stable, suggesting that the height with the higher capture capacity of the CBB for all periods was 0.5 m above the ground (see Table S1 and Figure 2 and Figure 3). A similar trend was observed in Exp B *χ^2^* = 10.13, *df* = 31, *P-value* = 0.001, except that the sampling date was not statistically significant (Table S1, Figure 2 and Figure 3).

We compared the capture rate of CBB traps placed at 1.5 m height from the ground in both CT and ST; see Figure S1 in both experiments. The results show the significance of *χ^2^* = 20.8, *df* = 22, *P-value* = 0.001 for Exp A. These findings suggest that the traps at 1.5 m in CT were more efficient at capturing CBB throughout the year than were the reference single traps (only one trap at 1.5 m, ST), see Figure S1A. Likewise, in Exp B, the traps at 1.5 m compared with ST showed significant differences of *χ^2^* = 10.85, *df* = 15, *P-value* = 0.001 (see Table S1 and Figure S1B). On the other hand, the infestation rate in the plots in Exp A and Exp B decreases over time. For Exp A, the initial infestation rate was 27.21% but ended up being 4.63%. For Exp B, the initial infestation rate was 3.81% and ended up as 1.77%. Nonetheless, in plot Ref 1, the infestation rate started at 20.23% and ended at 8.50%. For Ref 2, the infestation rate started at 10.18% and ended at 20.12%.

**Figure 4:**
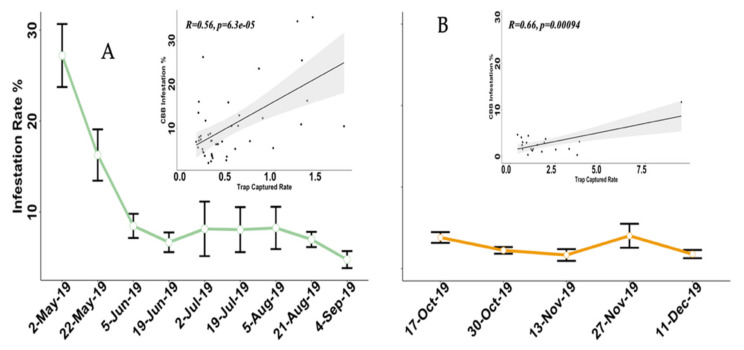
Plots of the average and standard error (vertical bar) of the infestation rate during experiments (**A**) and (**B**). The regression analysis shows a significant positive correlation between the percentage of infestation and trap capture. Experiment A was conducted from March 2019 to September 2019, and B was performed from October 2019 to March 2020, in Adjuntas, Puerto Rico.

The CBB infestation rate and trap capture rate of the CBB were higher earlier in Exp A. For Exp B, the trapping rate of CBB was higher at the end of the experiment. Results of the Spearman’s rank correlation test showed that there was a significant positive correlation between infestation in the coffee crop of the study plots and the CTs’ capture rate for Exp A and Exp B (*R* = 0.56 and *p*-value = 6.36 × 10^−5^ and *R* = 0.66 and *p*-value = 0.001; Figure 4). The slope of the regression analysis observed in Figure 4B is influenced by an extreme data point, resulting in limited use in this case. Additionally, the infestation rate of the single trap plot decreased over time (Figure S1 and Figure 4).

The results showed that independent of the season (sampling date) and coffee variety (‘Limani’ or ‘Catuaí’), the height that collected more CBB was 0.5 m above the ground. The total rain events (RE, Table 1) by the trapping period correlates with the total capture of CBB in Exp A; a significant difference is observed (*t* = 2.48, *df* = 10, *p*-value = 0.033). The other variables—total rain (*t* = 1.12, *df* = 10, *p*-value = 0.283), temperature (*t* = 2.05, *df* = 10, *p*-value = 0.074), and RH% (*t* = 2.52, *df* = 10, *p*-value = 0.44)—were not significantly different in either experiment. Nonetheless, for Exp B, there were no significant differences between CBB captured and environmental variables.

## 4. Discussion

Bait traps have been used worldwide, to monitor CBB populations’ flight on coffee plantations and then implement some sanitation strategies [1,3,8,13,14,16,18,19,26]. However, the significant number of CBB captured over time at the four different heights of the CTs in our study suggests that CTs themselves could be beneficial as a pest management control strategy (Figure S1). The height at which each trap is placed is crucial; our results establish a significant difference among the CT heights. It was the lowest height trap sites (0.5 m) that captured the highest numbers of CBB (Figure 2). Usually, to monitor CBB flight activity, the traps are used individually at 1.5 m above ground and not in columns [13,18,26]. Our results suggest that if the traps are placed at 0.5 m from the ground, their capacity to catch CBB throughout the year will be much higher than at the height they are commonly placed.

The berries remaining on the ground after the harvest are a potential refuge for the CBBs in the following months and in future coffee production. During this period (January to March), a significant number of the population of the CBBs are close to the ground [13,26]. Therefore, the height at which the trap is placed is critical in this period (Figure 3 and Figure S1). Our results support this idea; we found that a significant number of CBBs collected in this season probably came from the ground, i.e., from berries that fell to the ground at the harvest time (see Figure 3 and Figure S1). However, CT collected CBBs at all four heights during the entire studied periods with significant differences between the four heights over time. These results suggest that most of the CBB population has preferred a short or low flight. CBBs that survived in the dry berries (“raisins”) on the ground, when they fly again and depending on the availability of an attractant, will first colonize berries in branches closer to the ground. The decrease in the infestation rates of the plots where the CTs were installed could result from the increased capture by CTs (Figure S1). The use of CTs can help to explain and indicate earlier stages of CBB infestation more efficiently than STs, contradicting some previous assumptions that the ST would be the best option to monitor or control the CBB population [3]. Sprays targeting more dynamic periods of the pest could contribute to more effective control by properly targeting the adults [8]. The use of CTs replacing STs improves CBB capture, including in periods of low capture.

Regarding the environmental variables analyzed, we noticed a correlation between the rain events and total CBB captured in Exp A. The use of state-of-the-art automated-weather stations at these experimental sites increases the accuracy of the relationship between CBB capture and the considered environmental variables (RH, temperature, rain events). The continued acquisition and use of data throughout the seasons and different locations will potentially allow us to build better predictive patterns of CBB outbreaks and understand their relationship to environmental variables [2].

Both chemicals (ethanol and methanol) used in the trapping are heavier than the air. The CT design under normal conditions would produce an expanded zone of influence with an overall increase in attractiveness towards the lower areas. Our experiment also observed a positive column attraction in the CBB captures (four traps, four attractants by CT) as indicated in the results. This design would likely increase CBB capture in the lower height trap throughout the study period. However, different environmental conditions, the coffee crop stage, plant density, variety, and the crop cultivation system could influence the results. Additional work should investigate CT effectivity during young coffee plant stages.

## 5. Conclusions

CT is a useful element in indicating CBB infestation density and population distribution by height. Traps placed at a lower height always maintained the highest capture of CBB throughout the study period. Under the environmental conditions of this study, the traps with the maximum capacity to capture the CBB throughout the evaluation periods were those placed at 0.5 m from the ground. Further studies on column trapping design should be conducted to optimize and explore their use as a proper pest management tool.

## Figures and Tables

**Table 1:** Summary of environmental variables and total CBBs captured by the CTs at the study sites (Agricultural Experiment Station of the University of Puerto Rico, Adjuntas) showing the temperature (Temp °C), relative humidity (RH%), and total rainfall (R) on the sampling date. R = rain (mm), RE = rain events, DD = dry days.

Experiment A										
Date	Temp °C	Max	Min	RH%	Max	Min	R	RE	DD	CBB
3-Apr.-19	20.7 ± 4.4	30.5	12.1	85.1	100	44.7	52.0	11	8	7588
15-Apr.-19	20.8 ± 4.3	30.6	12.3	83.8	100	42.4	10.4	14	3	9563
2-May-19	21.2 ± 4.5	31.4	12.6	85.1	100	44.7	95.6	20	7	7577
22-May-19	22.2 ± 4.2	31.5	13.8	85.1	100	55.1	144.0	13	9	5092
5-Jun.-19	22.2 ± 3.6	31.3	16.1	89.1	100	55.6	84.6	29	13	1406
19-Jun.-19	23.2 ± 4.4	33.9	15.3	85.8	100	53.5	17.4	12	8	452
2-Jul.-19	23.5 ± 4.3	33.0	15.9	85.5	100	50.7	52.8	8	7	261
19-Jul.-19	23.0 ± 3.7	32.1	15.3	87.2	100	49.8	222.6	28	6	224
5-Aug.-19	23.5 ± 3.8	32.4	16.0	87.8	100	46.9	40.0	19	5	155
21-Aug.-19	23.7 ± 3.9	32.2	16.0	86.7	100	54.4	78.6	17	7	228
4-Sep.-19	23.5 ± 4.4	33.7	15.0	86.2	100	45.8	20.4	10	4	353
**Mean ± SD**	**22.5 ± 4.1**	**32.1**	**14.6**	**86.1**	**100.0**	**49.4**	**818.4**	**181.0**	**77.0**	**32,899.0**
**Experiment B**										
18-Oct.-19	21.8 ± 4.6	32.9	14.7	89.9	43.9	100	270.4	32	3	250
30-Oct.-19	22.0 ± 3.7	30.9	16.1	91.7	50.2	100	75.0	28	1	365
13-Nov.-19	21.9 ± 3.8	29.7	15.8	90.1	57.2	100	89.8	16	4	286
27-Nov.-19	20.9 ± 3.9	30.3	13.8	92.0	58.4	100	103.2	19	6	821
11-Dec.-19	21.1 ± 3.8	29.6	13.5	90.9	56.3	100	30.0	18	1	441
22-Jan.-20	20.8 ± 3.3	29.3	13.6	90.0	53.8	100	111.2	77	16	9574
5-Feb.-20	20 ± 4.2	29.7	12.2	91.5	50.1	100	79.2	24	4	11101
19-Feb.-20	21.6 ± 3.8	29.4	13.3	90.5	55.1	100	12.4	12	6	9368
4-Mar.-20	20.3 ± 4.1	29.7	12.7	89.1	42.2	100	95.4	30	6	7067
**Mean ± SD**	**21.2 ± 3.9**	**30.2**	**14.0**	**90.6**	**51.9**	**100.0**	**866.6**	**256.0**	**47.0**	**39,273.0**

## Supplementary Materials

The following are available online at https://www.mdpi.com/article/10.3390/insects12070607/s1, **Figure S1:** Comparison of traps placed at 1.5 m in the single (red) and in the column trap design (blue) showing the top insert, infestation rate of the coffee berries in the Experiment A and B plots (red) and berry infestation rate in the referential single trap plots (blue). **Figure S2:** Schematic design of traps in zigzag disposition in different selected coffee plots. The referential plot was the same for both experiments. Experiment A was carried out between March and September 2019 and Experiment B between October and March 2020 at the Agricultural Experiment Station in Adjuntas, University of Puerto Rico. **Table S1**: Analysis of variance of column traps in Experiment A and Experiment B.
