# Supplementary material for: Vertical Trapping of the Coffee Berry Borer, Hypothenemus hampei (Coleoptera: Scolytinae), in Coffee
Source: Insects. 2021 Jul 2;12(7):607. doi: 10.3390/insects12070607 (PMC8305300; doi:10.3390/insects12070607)
Supplement: Supplementary file 1 [file insects-12-00607-s001.zip › insects-1195103-supplementary.pdf]

Article

Vertical Trapping of the Coffee Berry Borer, *Hypothenemus hampei* (Coleoptera: Scolytinae), in Coffee

Claudia Patricia Ruiz-Diaz and José Carlos Verle Rodrigues *

Supplementary Materials

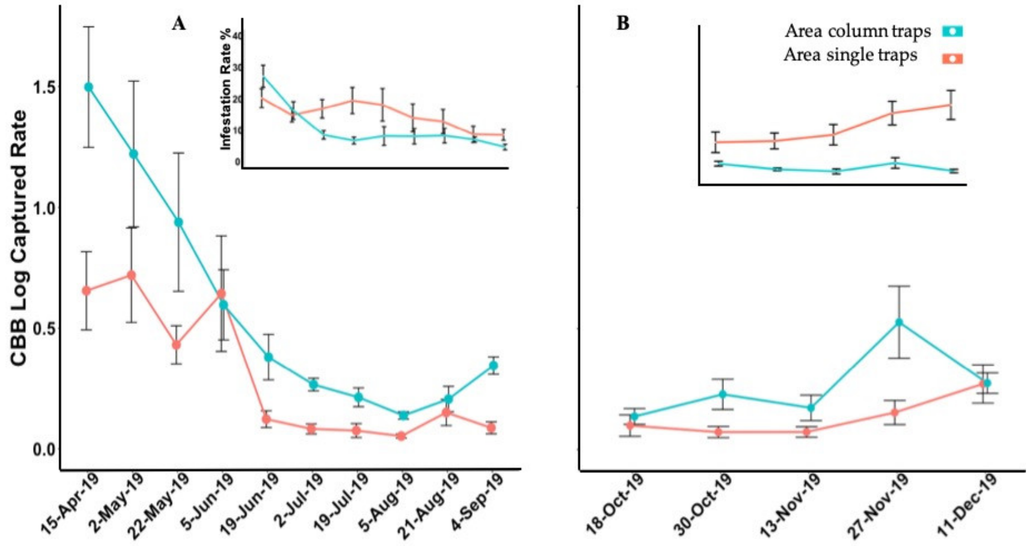

Figure S1: Comparison of traps placed at 1.5 m in the single (red) and in the column trap design (blue) showing the top insert, infestation rate of the coffee berries in the Experiment A and B plots (red) and berry infestation rate in the referential single trap plots (blue).

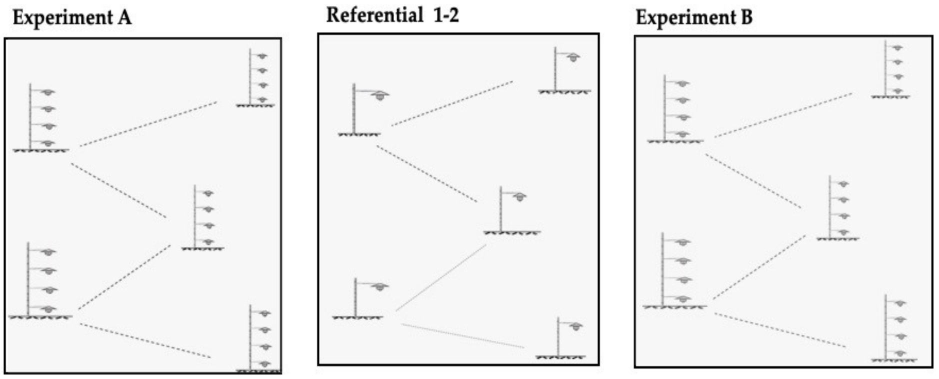

Figure S2: Schematic design of traps in zigzag disposition in different selected coffee plots. The referential plot was the same for both experiments. Experiment A was carried out between March and September 2019 and Experiment B between October and March 2020 at the Agricultural Experiment Station in Adjuntas, University of Puerto Rico.

Table S1: Analysis of variance of column traps in Experiment A and Experiment B.

	df	Pr(>Chi)
**Experiment A**		
	df	Pr(>Chi)
Trap Height	*3*	$<2.2e^{-16}$ ***
Date Sampler	*10*	$7.867e^{-11}$ ***
Trap Height: Date Sample	*30*	$< 2.2e^{-16}$ ***
**Experiment B**		
Trap Height	*3*	$< 2.2e^{-16}$ ***
Date Sampler	*7*	*0.0814435*
Trap Height: Date Sample	*21*	*0.0001252* ***
